# Perceived family cohesion, social support, and quality of life in patients undergoing treatment for substance use disorders compared with patients with mental and physical disorders

**DOI:** 10.1186/s13722-021-00252-8

**Published:** 2021-06-30

**Authors:** Bente Birkeland, Bente Weimand, Torleif Ruud, Darryl Maybery, John-Kåre Vederhus

**Affiliations:** 1 Department of Psychosocial Health, University of Agder, Grimstad, P.O. Box 422, N-4604 Kristiansand, Norway; 2 Faculty of Health and Social Sciences, University of South-Eastern Norway, Drammen, Norway; 3 Division of Mental Health Services, Akershus University Hospital, Lørenskog, Norway; 4 Institute of Clinical Medicine, University of Oslo, Oslo, Norway; 5 Monash Rural Health, Monash University, Warragul, Australia; 6 Addiction Unit, Sørlandet Hospital, Kristiansand, Norway

**Keywords:** Family cohesion, Social support, Quality of life, Substance use disorders, Norway

## Abstract

**Purpose:**

Support from family and other social network elements can be important in helping patients to cope with practical and emotional consequences of diseases. The aim of the study was to examine perception of family and social support and quality of life (QoL) in patients undergoing treatment for substance use disorders (SUDs). We compared them with patients in treatment for mental disorders (MDs) and physical disorders (PDs).

**Methods:**

We used data from a national multicenter study that recruited patients (N  =  518) from three treatment domains; SUD treatment units, MD treatment units, and PD treatment units (severe neurological conditions or cancer). Data on family cohesion, social support, and QoL were compared across patient groups. In addition, data on health variables was collected. We used a multiple linear regression procedure to examine how health and support variables were associated with QoL.

**Results:**

Family cohesion and social support in the SUD and MD groups were rated at similarly low levels, substantially lower than in the PD group. The SUD group exhibited a somewhat lower QoL than did the PD group, but their QoL was still in the near-to-normal range. In contrast, the MD group had markedly low QoL. When examining factors associated with QoL, we found that greater family cohesion and social support were positively associated with QoL. Mental distress was the strongest factor, and was negatively associated with QoL (beta − 0.15, 95% CI  =  − 0.17/− 0.14, p  <  0.001).

**Conclusion:**

Service providers need to be aware of the weaker networks and less regulatory family and/or social support available to patients with SUDs. Providers should focus consistently on the social networks of patients and include patients’ families in treatment processes.

## Background

A substance use disorder (SUD) can be a serious clinical condition causing major health problems and affecting a wide range of life domains [1, 2]. In times of illness, the family is a primary source of support for patients [3, 4]. This support includes facilitating patients’ adaptation to living with the illness, improving their compliance with treatment, and in that way promoting recovery [5]. Thus, family cohesion has been considered a buffer against drinking and substance use, and a significant protective factor of substance abuse relapse among populations with such problems [6, 7]. Conversely, poor levels of family cohesion are related to greater levels of drinking and substance use [8]. However, when an SUD exists within a family, there is a risk that the strain the family experiences will lead to exhaustion or broken relationships [9, 10]. Efforts to support the family in order to enhance cohesion among family members, would thus seem beneficial both to the patient and to the family as a whole [7, 11]. Support from networks beyond the family (e.g., peer support, support groups, self-help groups) may be an additional resource to help patients cope with practical and emotional consequences, as well as in maintaining remission [12, 13]. Higher level of social support, defined as being socially connected [14], has been associated with reduction of substance use and improved mental health for persons experiencing an SUD [15–17].

The aim of the present study was to examine patients’ own perception of family cohesion and social support. To put findings from the SUD treatment field into perspective, we compared SUD patients with patients from two other patient groups: those with mental disorders (MDs) and those with physical disorders (PDs). Family cohesion and social support are relevant to recovery, as described above, and we are not aware of any study that has examined these support factors across several patient groups including patients with SUDs. The rationale for the comparison was that family cohesion and social support is said to be equally important for patients with MDs. Stronger network support has the potential to reduce relapse and hospital admissions, encourage compliance with medication and, of direct relevance here, to reduce social impairment and improve general functioning [18, 19]. Positive change in perceived social support is recognized as a mediator of change in subsequent depressive and anxious symptomatology, and a higher level of social support has been associated with symptom reduction in patients with MDs [19, 20]. Thus, lack of family cohesion and social support would make patients with MDs and SUDs vulnerable in the recovery phase. Due to the often sudden onset of physical problems (e.g., cancer diagnosis) we considered the PD group likely to resemble more those of the general population in terms of the familial and social situation. We hypothesized that patients admitted to SUD treatment units would rate family cohesion and general social support at least as low as the MD group and considerably lower than those admitted to PD treatment units.

As a main dependent or “outcome” variable in our analyses, we used the concept of quality of life (QoL); an overarching construct of health and well-being, considered to be a general aim across different patient groups and disorders [21, 22]. Thus, we aimed to examine how support variables were associated with QoL across patient groups. Among people experiencing an SUD, studies have reported significantly lower QoL compared with the general population [23]. Furthermore, substance abuse and/or mental distress is associated with low QoL [9, 24, 25]. For the present analysis, we expected that the SUD and MD groups would exhibit the lowest QoL and that greater family cohesion and social support would be positively associated with QoL.

## Methods

### Design, study setting, participants and procedures

This study used data from a cross-sectional, multicenter study, conducted in five Norwegian hospitals, in which the overall objective was to explore the experience of children when one of their parents had an illness, as well as the family’s perceived need for support [26]. In accordance with the overall aim of the study, the inclusion criteria was that the patient provided parental care for children under age 18. We approached patients in three illness domains within the Norwegian specialist health care services and recruited 129 in SUD treatment units, 194 in MD units, and 195 in PD units (severe neurological conditions or cancer). There were no exclusion criteria other than not being able to understand the Norwegian language to complete the questionnaire. The present study examined the patients’ own perception of family cohesion and social support, as the patient gave information about their family situation, and reported on their own health and life situation. Data collection took place from March 2013 to December 2014. Trained personnel such as health care or social workers recruited patients and collected the data. The data collection took place according to participants’ choice, usually at their home. The data were collected on digital tablets and the total questionnaires required about 1 h to complete. The digital data strategy ensured that questionnaires were completed in full.

### Measures

Basic demographic variables were collected, including age, gender, education, income, and occupation status. Occupation was defined as any positive occupational activity and included full-time and part-time occupations or ongoing education.

#### Family and social variables

Data was collected about partner status, that is, whether patients were living alone or with a partner. To assess family support, we used the Family Cohesion Subscale in the Family Adaptability and Cohesion Evaluation Scales (FACES-III). The scale assesses the emotional bonding family members have towards one another and the perceived connectedness within the family and, as such, it serves as a proxy for measuring emotional support in a family [27, 28]. The instrument consists of 10 statements, for example, “family members ask each other for help”, and were rated on ordinal scales from 1 (almost never) to 5 (almost always). The sum score ranged from 10 to 50 and higher scores indicated higher perceived cohesion. The Interpersonal Support Evaluation List (ISEL) was used to measure the perceived availability of social support [29]. ISEL has 12 statements about social support rated on an ordinal scale (e.g., “I feel that there is no one I can share my most private worries and fears with”), with responses ranging from 1 to 4 (“definitely true”, probably true”, “probably false”, and “definitely false”). Ratings of positively worded items were reversed and a higher score indicated a more positive evaluation of social support. The sum score of the scale ranged from 12 to 48.

#### Health and treatment variables

We used an adapted version of the *CAGE-AID* (including both alcohol and drugs), to assess the lifetime prevalence of substance use problems [30]. A sum score based on four questions was calculated (range 0–4) and a score  ≥  2 was the cut-off for a substance use problem. The Hopkins Symptom Checklist (HSCL-10) was used to assess mental distress [31]. Patients rated the ten items on a 4-point scale (1–4) and an average was computed to indicate a global severity index (GSI). Higher scores indicate greater distress and the cut-off point for pathology was 1.85. The illness was described with questions about illness duration (years since illness debut) and perceived prognosis, that is, whether there was uncertainty about how the illness would develop in the future. We also collected information about treatment duration for the current condition.

#### Quality of life

We used the QoL-5 to assess QoL. The QoL-5 is a non-disease-specific scale, suitable for assessing QoL across different treatment domains. Patients rated their perceived physical and mental health, and social situation, that is, their relationships with their partner and friends, and relationship with themselves, described as existential QoL by the developers [32]. Responses were scored on a 5-step ordinal scale from 1 to 5 and transposed to a decimal scale ranging from 0.1 to 0.9, where 0.9 was the best and 0.1 was the worst possible rating [33]. The mean score of the scale is an expression of global QoL. Normative data from a Danish general population survey indicated that the general population norm was a mean score of 0.69 [34]. Population norms for each item on the scale do not exist, but the overall norm (~  0.7) has been used in previous research [23]. A score  ~  0.15 below that of the general population has been suggested to represent a clinically significant reduction in QoL, and scores below this cut-off (i.e.,  <  0.55) represent a markedly reduced QoL [35]. The internal consistency of the scale is good; the composite reliability coefficient was 0.87 in previous research [36].

### Statistics

We used descriptive statistics to present socio-demographic variables. Differences between groups in family, social, and health variables were examined using the chi-square test for categorical variables and ANOVA for continuous variables. If significant, we continued with pairwise tests (chi-square or Student’s t test). A multiple linear regression with simultaneous entry of variables was used to examine how the health and support variables were associated with QoL. We also controlled for demographic variables in this analysis. Results have been presented with standardized and non-standardized beta coefficients (β) with 95% confidence intervals (CIs). The adjusted R square (R^2^) value assessed the percentage of the variation in QoL explained by the model. Preliminary analyses were undertaken to check for multicollinearity among the independent variables. None of the included variables had a Variable Inflation Factor (VIF) value higher than the recommended cut-off, i.e.,  ≤  3 (all VIF values were below 2.1) [37, p. 316]. The significance level was set at p  <  0.05. We used IBM SPSS version 25 for analyses [38].

## Results

Patients (N  =  518) had a mean age of 38 years and 69% were female (Table 1). The SUD and MD group were 7 years younger (35 versus 42 years) and had a substantially lower educational level than the PD group. The same trend applied to occupation and income, with substantially lower occupational levels and incomes than the PD patients. The educational level and income were especially low in the SUD group, with only 17% having education above the high school level and with 60% lower income than the PD group. Only four of ten in the SUD group were women, while the majority in the two other groups, approximately eight of ten, were female (Table 1).

**Table 1:** Characteristics of participants (N  =  518)

Variables	PD N = 195	MD N = 194	SUD N = 129	Total N = 518
Age, years, mean (SD)	42 (7)	35 (9)	35 (8)	38 (8)
Gender, women, N (%)	143 (73)	162 (84)	53 (41)	358 (69)
Educational level, N (%)				
Primary education	20 (10)	30 (16)	52 (40)	
High school	69 (35)	97 (50)	55 (43)	221 (43)
College/university	106 (54)	67 (35)	22 (17)	195 (38)
Work or studying^a^, N (%)	117 (60)	79 (41)	52 (40)	248 (48)
Income^b^, mean (SD)	930 (1064)	599 (626)	383 (243)	670 (796)
Treatment duration, months, median (IQR)	8 (4–36)	16 (5–48)	19 (6–41)	13 (5–42)

*PD* physical disorders group; *MD *mental disorders group; *SUD *substance use disorder group; *SD *standard deviation, *IQR *interquartile range

^a^Proportion with at least some work/school occupation

^b^Income in 1.000 Norwegian currency; kroner (NOK)

### Family cohesion and social support

Related to the main aim of the study, the SUD and MD groups rated their family cohesion and social support to be significantly lower than the PD group (Table 2). A substantially higher proportion were living without a current partner; 38% and 26% in the SUD and MD group, respectively, versus only 13% in the PD sample. Compared with the PD sample, the SUD and MD groups had, respectively, 4.0- and 2.6-point lower scores on the family cohesion scale. The perceived social support score (ISEL) was a substantial  ~  4 points lower in the SUD and MD groups compared with the PD sample (Table 2).

**Table 2:** Family and social support, health variables and QoL (N  =  518)

Variables	PD N = 195	MD N = 194	SUD N = 129	*p* value^a^	PD/MD^b^	PD/SUD^b^	MD/SUD^b^
No partner, N (%)	25 (13)	57 (29)	49 (38)	< 0.001	< 0.001	< 0.001	ns
Family cohesion sum score^c^, mean (SD)	42.8 (5.8)	40.2 (7.8)	38.8 (8.9)	< 0.001	< 0.001	< 0.001	ns
Social support sum score^d^, mean (SD)	40.1 (6.7)	35.5 (7.4)	36.1 (7.3)	< 0.001	< 0.001	< 0.001	ns
Problematic substance use^e^ (in lifetime), N (%)	6 (3)	27 (14)	72 (56)	< 0.001	< 0.001	< 0.001	< 0.001
Mental distress^f^, mean (SD)	1.71 (0.58)	2.45 (0.74)	1.89 (0.71)	< 0.001	< 0.001	< 0.05	< 0.001
Future uncertainty about the illness, N (%)	160 (82)	141 (73)	45 (35)	< 0.001	< 0.05	< 0.001	< 0.001
Duration of the illness (years), mean (SD)	4.5 (9.7)	10.3 (9.9)	13.0 (11.5)	< 0.001	< 0.001	< 0.001	< 0.05
Quality of life (QoL)^g^, mean (SD)	0.66 (0.14)	0.50 (0.16)	0.62 (0.17)	< 0.001	< 0.001	< 0.05	< 0.001

*PD *physical disorders group, *MD *mental disorders group, *SUD *substance use disorder group

^a^Overall p value for difference between groups obtained from the chi-square test for categorical and ANOVA test for continuous variables

^b^p value for pair-wise tests were obtained from the chi-square test or Student’s t test

^c^Family cohesion was measured with the Family Adaptability and Cohesion Evaluation Scales (FACES-III)

^d^Social support was measured with the Interpersonal Support Evaluation List (ISEL)

^e^Problematic substance use was measured with the CAGE-AID

^f^Mental distress was measured with the Hopkin’s Symptom Check List-10—score 1–4

^g^Quality of Life was measured with the QoL-5, score 0.1–0.9

### Health variables and QoL

Concerning health variables, the proportion with problematic substance use was much higher in the SUD group; 56% versus 14% and 3% in the other two groups (Table 2). Perceived mental distress was much higher in the MD group versus the two other groups, and the MD group had a mean score considerably above the clinical cut-off. The majority of the PD sample and the MD group reported uncertainty about the future (82% and 73% respectively), while a minority (35%) did in the SUD sample. In contrast, the longevity of the illness was greatest among the patients with SUDs: 13.0 years compared with 10.3 and 4.5 years in the MD and PD groups, respectively (Table 2).

**Fig. 1:**
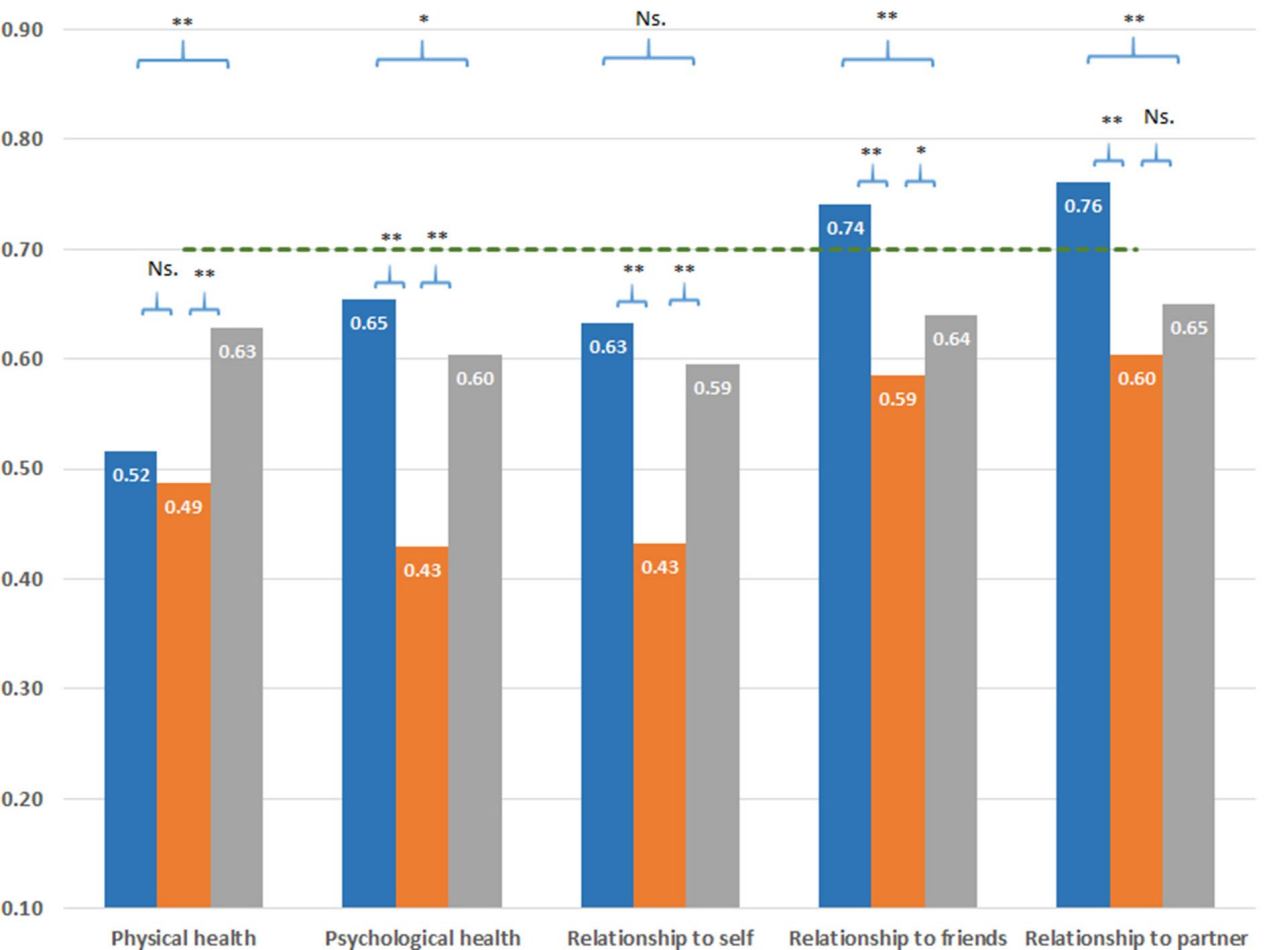
Comparison of scores on each QoL item of the QoL-5 measure across groups. Notes: the overall p value was  <  0.001 for all constructs (ANOVA test). p values from pair-wise tests were obtained using Student’s t test. *  ≤  0.05, **  ≤  0.001, *Ns.* not significant. Blue column  =  physical disorders group. Orange column  =  mental disorders group. Grey column  =  substance use disorder group. Green line  =  population mean

Only the MD group had a mean QoL score in the markedly low range (0.50; Table 2). When reporting QoL in detail, the PD group rated their physical QoL as markedly reduced (0.52), but otherwise they had normal or near-to-normal QoL scores (Fig. 1). Except for physical QoL, the SUD group rated their QoL as somewhat lower compared with the PD patients, but still in the near-to-normal range. In the MD group, however, QoL was markedly lower compared with the other two groups, especially for ratings of psychological and existential QoL (0.43, Fig. 1). The MD sample even rated their physical QoL on a similarly low level as those admitted for PDs.

### Associations with QoL

Unexpectedly, treatment domain did not surface as a significant explanatory factor for variation in QoL in the multiple regression analysis (Table 3), indicating that factors across treatment domains are more relevant to explain variation in QoL. Only three variables were significantly associated with QoL. Higher social support (beta 0.05, 95% CI 0.03/0.06, p  <  0.001) and family cohesion (beta 0.03, 95% CI 0.02/0.04, p  <  0.001) were positively associated with QoL. Mental distress was negatively associated with QoL: beta − 0.15 (95% CI − 0.17/− 0.14, p  <  0.001), and was the strongest influencing factor as evidenced by the highest standardized beta (Table 3). The model explained 65% of the variation in QoL.

**Table 3:** Factors associated with QoL (N  =  518)

	Beta (95% CI)^b^	Standardized beta	*p*
Sociodemographic variables			
Patient group	0.00 (− 0.02/0.02)	0	0.918
Gender	0.00 (− 0.02/0.02)	0.01	0.709
Age	0.00 (0.00/0.00)	− 0.01	0.619
Education	0.00 (− 0.01/0.01)	0	0.912
Having a partner	0.00 (− 0.02/0.02)	− 0.01	0.808
Work or studying	0.01 (− 0.01/0.03)	0.03	0.291
Income	0.00 (0.00/0.00)	− 0.03	0.336
Social support variables^a^			
Social support (ISEL)	0.05 (0.03/0.06)	0.17	< 0.001
Family cohesion (FACES)	0.03 (0.02/0.04)	0.13	< 0.001
Health and treatment variables			
Duration of the illness	0.00 (0.00/0.00)	0.03	0.313
Uncertainty about the future	− 0.02 (− 0.04/0.01)	− 0.04	0.171
Treatment duration	0.00 (0.00/0.00)	0.04	0.192
Problematic substance use	0.00 (− 0.02/0.03)	0.01	0.871
Mental distress	− 0.15 (− 0.17/− 0.14)	− 0.68	< 0.001

*FACES* Family Adaptability and Cohesion Evaluation Scales; *ISEL *Interpersonal Support Evaluation List

^a^In this analysis, we used the mean scores of FACES and ISEL to facilitate interpretation

## Discussion

Family cohesion was lowest in the SUD and MD groups, and these two groups also rated their social support at a similarly low level, substantially lower than did the PD sample. The QoL score of the MD group was markedly lower than in the PD sample. In contrast, the SUD sample exhibited a somewhat lower QoL than did the PD sample, but their QoL was still above the cut-off for a markedly low QoL. When examining factors associated with QoL, mental distress came out as the strongest factor, and was negatively related to QoL.

A main aim of this study was to determine differences between patients with SUDs compared to MDs and PDs in their perceptions of family cohesion, social support, and QoL. While family and extended social support networks can be helpful in improving substance use outcomes, reducing mental distress, and supporting recovery [15, 39], it is thought that the longer-term strain and broken relationships might ‘test’ the ongoing level of support from the family and/or social network [9, 10]. The current data support this notion, with lower levels of family cohesion and social support for the MD and SUD groups. The strain and broken relationships might be illustrated in the current data with significantly longer duration of the illness (approximately three times longer for the SUD group compared with the PD group) and more patients in the SUD and MD groups having no partner. It has been observed that SUDs in a family have a disruptive effect on the functioning of a family and, as the illness progresses, it is followed by a decline in the quality of family relationships [10]. For a patient with an SUD, this is doubly lamentable, as it means that positive familial restraining influences may no longer be present and there may be a lack of motivational support to promote necessary behavioral changes in the patient.

As expected, we observed more problematic substance abuse in the SUD group, higher mental distress in the MD group, and lower physical QoL score ratings for the PD group. Unexpectedly, the SUD group exhibited near-to-normal physical health, while there was a very low physical health rating in the MD group, equally low as in the PD group, as represented by the physical QoL score. Low patient-reported rating of physical health among persons with MDs has also been observed in previous research. A large European study found that patients with many categories of mental disorders, for example, dysthymia, any mood disorders and post-traumatic stress disorder, rated their physical health on a similarly low level as people with chronic physical diseases like diabetes, lung disease, and arthritis [40]. Physical health ratings in SUD treatment studies are inconsistent. A large European epidemiological study found near-to-normal reported physical health among patients with SUDs [40], while a previous Norwegian study of patients with severe SUDs admitted to a detoxification (detox) center reported similarly low physical health among patients with SUDs as inpatients admitted to a general medical ward [23]. The patients with SUDs in that study had also low physical health ratings similar to the MD group of the present study. Thus, it is likely that the severity level of the SUD will influence rating of physical health.

The overall QoL score of the SUD group was close to the normal range and was only slightly lower than that seen in the PD group. This is unexpected in light of previous studies reporting substantially lower QoL among patients with SUDs compared with normative populations and compared with patients admitted to general medical wards [23, 24, 41]. The relatively high QoL level in the SUD group is also puzzling in light of the familial and social strain reported by this group. In contrast, the MD group reported a markedly low QoL, which represented a clinically significant reduction in QoL according to the interpretative guidelines [35]. We note here that the present respondents had already been in treatment for a substantial period (median 19 and 16 months for the SUD and MD group, respectively). Although we do not have data from their treatment admission, it is possible that their QoL has improved more with time than that of the MD group or, alternatively, that the MD group started at an even lower QoL at admission.

When examining associations with QoL, we found that family cohesion and social support related positively to QoL, as hypothesized, while elevated mental distress was negatively and strongly related to QoL: a one-point higher mental distress score resulted in a substantial 0.15 lower QoL score. From a predictive perspective, previous studies of SUD treatment have found that worse mental health at baseline also predicted worse QoL at a later follow-up [42]. Seen from the perspective of the patients with SUDs, it is important to pay attention also to their mental health, as it is widely accepted that an SUD combined with a comorbid MD and/or elevated levels of mental distress can have a negative impact on the patient’s condition and functioning [43]. Thus, although the mean level of mental distress among the SUD group in the present study was only slightly above the clinical cut-off, it is important to also follow up on their mental health [44].

Based on our study, we propose further research on family cohesion and social support in families with parental substance use disorder. We specifically recommend developing models for clinical support and guidance for families related to better family cohesion and increased social support. Furthermore, we recommend implementation studies with fidelity- and effect measurements.

### Methodological considerations

The usual caveat about the interpretation of causality in cross-sectional research must be kept in mind: using this design, we cannot determine whether the independent variables caused the variation in QoL. The inclusion of patients was based on the treatment service the patient was admitted to, not on their diagnosis. Thus, we cannot rule out that some patients might have had diagnoses that would make them eligible for inclusion in another patient group.

There are some indications in the data that could lead to speculation about whether the SUD group consisted of patients with a less severe SUD. These include the relatively high QoL score and the fact that the CAGE-AID detected only 6 of 10 in the SUD group as having an SUD. There was also a low proportion of the SUD group who reported uncertainty about the future development of the condition. However, the longevity of the SUD patients’ problems argues against their having less severe SUDs. There is also a health system argument against the assumption of a mild SUD: to get access to specialized SUD treatment services in Norway, one has to present with at least a moderately severe SUD. The unexpectedly high QoL in the SUD group might be related to the assessment being performed in the midst of a treatment period combined with the relatively high optimism in the SUD group. The latter might indicate that this group had higher prognostic optimism than patients with MDs or PDs. Being in SUD treatment may have brought about a higher expectation of improvement and hope of recovery than in the other two groups. Alternatively, there might have been different selection bias across groups. The research assistant indicated that the families with the most difficult and challenging care situation were less likely to participate and this was more noticeable for patients and families recruited from the MD and SUD treatment units. Thus, the differences between patient groups would have been even larger in disfavour of the MD and SUD groups if this selection bias could have been avoided.

## Conclusions

The present study puts social support and family cohesion in the SUD treatment field into perspective when compared with other treatment fields. When having an SUD, it is vital to build and maintain positive relationships to protect oneself from the influences of negative relationships [45]. To improve outcomes for patients with an SUD, it is important to be aware of the weaker network and less regulatory family and/or social support available to these patients. Service providers therefore need to have a consistent focus on the social networks of patients and include their families in treatment processes.

## Data Availability

The data used in this study form the basis of ongoing PhD and postdoctoral studies. The data will be anonymized on Dec 31, 2021, after which, according to current Norwegian regulations and practice, it can be deposited in a publicly available data repository. An anonymized dataset used in the current study is available from the corresponding author upon reasonable request.

## Abbreviations

CAGE-AID: Cut down, annoyed, guilt, eye opener (screening tool for alcohol and substance use problems)
FACES: Family Adaptability and Cohesion Evaluation Scales
HSCL: Hopkins Symptom Checklist
ISEL: Interpersonal Support Evaluation List
MD: Mental disorder
PD: Physical disorder
QoL: Quality of life
SUD: Substance use disorder
